# HDAC6 deficiency exacerbates atherosclerosis via STAT3-K685 acetylation-mediated CD36/SR-A upregulation in macrophages

**DOI:** 10.1038/s41419-025-08344-y

**Published:** 2025-12-24

**Authors:** Wenqing Wang, Yue Jiang, Xuan Pan, Dong Chen, Hui Yang, Wang Pan, Mingjie Pan, Bin Wang

**Affiliations:** 1 Clinical Stem Cell Center, Nanjing Drum Tower Hospital, Affiliated Hospital of Medical school, Nanjing University, Nanjing, PR China; 2 Joint Institute of Nanjing Drum Tower Hospital for Life and Health, College of Life Science, Nanjing Normal University, Nanjing, PR China

**Keywords:** Atherosclerosis, Acetylation

## Abstract

Atherosclerosis (AS) is a prevalent chronic arterial disease characterized by excessive cholesterol accumulation in the arterial intima. While substantial progress has been made in elucidating its risk factors and pathogenesis, the upstream signaling molecules that drive the initiation and progression of AS remain poorly understood. Analysis of monocyte samples from the GSE23746 database revealed that Histone Deacetylase 6 (HDAC6) expression was significantly downregulated in patients with carotid atherosclerosis compared to healthy controls. In vitro experiments further demonstrated that HDAC6 deficiency markedly promotes foam cell formation in macrophages, a process dependent on its deacetylase activity. Mechanistically, HDAC6 interacts with signal transducer and activator of transcription 3 (STAT3) and regulates its acetylation at K685, a critical modification that facilitates macrophage foam cell formation. Specifically, the loss of HDAC6-mediated deacetylation leads to increased STAT3-K685 acetylation, which in turn upregulates the expression of CD36 and SRA, thereby enhancing cholesterol uptake in macrophages. Our findings establish HDAC6 as a protective regulator in atherosclerosis, which maintains lipid metabolic homeostasis by modulating the STAT3-CD36/SR-A axis. We also observed that systemic HDAC6 knockout exacerbated atherosclerotic progression in high-fat diet-fed *ApoE*^⁻/⁻^mice, accompanied by increased monocyte/macrophage infiltration into plaques. Collectively, this study establishes HDAC6 as a potential therapeutic target for atherosclerosis intervention.

## Introduction

Atherosclerosis (AS), a prevalent arterial disease, is characterized by dysregulated lipid metabolism, inflammation, and excessive cholesterol accumulation in the arterial intima [1–3]. In this condition, monocytes are recruited to counteract the buildup of apolipoprotein B-lipoproteins in the matrix beneath the endothelial layer. Upon recruitment, monocyte-derived macrophages engulf large amounts of lipids, transforming into foam cells [4]. This foamy transformation of monocyte-derived macrophages represents one of the most critical events in the initiation and progression of atherosclerosis [5, 6]. The underlying mechanism involves macrophages taking up oxidized low-density lipoprotein (ox LDL) via scavenger receptors, such as CD36 and scavenger receptor A (SR-A), located on the cell membrane surface, thereby converting them into foam cells [7]. Studies have demonstrated that the number of macrophages, primarily derived from circulating monocytes, can increase by up to 20-fold within the mouse aortae during atherosclerosis [8].

Although numerous studies and advances have been made in understanding the risk factors and pathogenesis of AS, significant confusion remains regarding the mechanisms and roles of epigenetic modifications in its onset and progression [9, 10]. Acetylation and deacetylation, regulated by histone acetyltransferases (HAT) and histone deacetylases (HDACs), are critical post-translational modifications in cells. These processes influence protein biological activity and the mammalian metabolome in multiple ways [11]. It has been reported that activated P300, one of HATs, contributes to atherogenesis [12]. Furthermore, inhibition of P300 by its specific inhibitor C646 reduces lipid droplet accumulation in macrophages, suggesting a potential protective role against atherosclerosis [13]. In addition to HATs, HDACs consist of at least 18 members in mammals, categorized into four classes [14]. Class I HDACs, such as HDAC1/2/3/8, primarily rely on Zn^2+^ as a cofactor. Class IIa HDACs, including HDAC4/5/7/9, also depend on Zn^2+^, but exhibit distinct cellular localization and functions compared to Class I. Class IIb HDACs, including HDAC6/10, are independent of Zn^2+^ and possess two catalytic domains. Class IV contains only HDAC11, an NAD^+^ dependent enzyme that does not require Zn^2+^.

Previous studies have revealed complex and often divergent roles of different HDAC classes in atherosclerosis. For instance, HDAC1 promotes artery injury through activation of VAV3 by binding to miR-182-5p in atherosclerotic mice model [15], while myeloid HDAC2 deficiency in high-calorie diet-fed *LDLR*^−/−^ mice reduced atherosclerosis in males without altering systemic metabolic parameters[16]. The lncRNA Kcnq1ot1 was shown to accelerate atherosclerosis in *ApoE*^−/−^ mice by upregulating HDAC3, thereby suppressing ABCA1 expression and cholesterol efflux in macrophages [17]. In contrast, HDAC4 activation by Krüppel-like factor 7 (KLF7) attenuated atherosclerotic lesions and improved metabolic abnormalities in mice [18]. HDAC5 binds and inhibits the atheroprotective transcription factor KLF2, potentially promoting endothelial dysfunction and atherosclerosis [19]. HDAC7 is upregulated in atherosclerosis and regulates proinflammatory genes such as IL-6 and MCP-1 [20]. Similarly, HDAC9, another Class IIa member, is widely recognized as a genetic risk factor for atherosclerosis in both humans and mouse models [21]. Pharmacological inhibition studies further highlight this complexity: the HDAC1/2 inhibitor Romidepsin epigenetically suppresses VCAM-1 expression and attenuates atherosclerosis [22]; the HDAC3-specific inhibitor RGFP966 alleviates lesion formation and EndMT in plaque of *ApoE*^−/−^ mice [23]; and TMP195, a selective class IIa (HDAC4/5/7/9) inhibitor, significantly reduces atherosclerosis progression and inflammation [24]. However, Trichostatin A (TSA), a pan-HDAC inhibitor of Class I and II HDACs, unexpectedly increased plaque size in a mouse model of atherosclerosis [25], suggesting that HDAC inhibition may have divergent effects depending on the target subclass. In particular, these findings imply that class IIb HDAC members may exert distinct roles in atherogenesis though their direct functions remain poorly understood. Recent research indicates that HDAC6 negatively regulates lipid droplet fusion in adipocytes and lipogenesis in non-alcoholic fatty liver disease [26, 27]. In view of the crucial role of lipid metabolism in atherosclerosis, exploring the role of HDAC6 in lipid metabolism and its impact on the AS holds significant research value for developing more precise therapeutic target.

In this study, we retrieved genomic expression profiles of atherosclerosis cases and matched controls from the Gene Expression Omnibus (GEO) database. Our analysis revealed that HDAC6 expression was significantly lower in patients with carotid atherosclerosis compares to normal controls. Subsequently, in vivo experiments showed that systemic *HDAC6* knockout (*HDAC6*^*−/−*^) increased macrophage infiltration into atherosclerotic plaques and worsened atherosclerotic development in *ApoE*^−/−^ mice with a high-fat diet. In vitro, either HDAC6 deficiency or treatment with an HDAC6-specific inhibitor suppressed its deacetylase activity, which markedly promoted the formation of macrophage-derived foam cells. This effect was mediated by enhanced acetylation at K685 and phosphorylation of signal transducer and activator of transcription 3 (STAT3), leading to increased cholesterol uptake in macrophages and upregulated CD36 and SR-A expression. In conclusion, our study demonstrates that HDAC6 acts as a regulator of lipid metabolic homeostasis in atherosclerosis, thus providing a potential new target for the prevention and therapeutic intervention in atherosclerosis.

## Materials and methods

### The analyze of GEO databases

We downloaded the expression data of atherosclerosis samples from GEO databases. The differentially expressed genes were calculated by Student's *t* test. To evaluate the association between the gene expression and survival, we categorized the samples into two group by median of individual gene expression. The *P* value of trend was generated by the Cox regression model. Kaplan–Meier survival curves were drawn and compared among subgroups using Cox proportional hazard model. All statistical analyses were performed using R version 4.4.0 (The R Foundation).

### Animal experiments

The *apolipoprotein E* (*ApoE*) knockout (*ApoE*^−/−^) mice on the C57BL/6 background ([T001458] B6/J^Gpt^-Apoe^em1^Cd^82/Gpt^) were purchased from Model Animal Research Center of Nanjing University (Jiangsu, Nanjing, China). The *HDAC6* knockout (*HDAC6*^−/−^) mice on the C57BL/6 background were a generous gift of Professor Tso-Pang Yao’s Lab (Duke University, USA). The breeding environment is a specific pathogen-free animal facility.

*HDAC6*^−/−^ mice and *ApoE*^−/−^ mice were bred to generate *ApoE*^−/−^/*HDAC6*^−/−^ mice. DNAs from mouse tails were extracted by the One Step Mouse Genotyping Kit (Cat#PD101; Vazyme; Nanjing, China). And the mouse genotyping was performed by PCR with the primer sequences to flank the targeted *ApoE* gene and *HDAC6* gene sequence, respectively. The primer sequences and PCR program are shown in Supplementary Tables 1 and 2 of the Supplementary materials, respectively.

Eight-week-old male WT (*n* = 10), *HDAC6*^*−/−*^ (*n* = 10), *ApoE*^−/−^ (*n* = 14), and *ApoE*^−/−^/*HDAC6*^−/−^ (*n* = 14) mice were fed with high-fat diet (HFD, containing 40% fat and 1.25% cholesterol) and free access to water ad libitum for 12 weeks. The body weight of the model mice was recorded from the beginning to sacrifice day. The genotyped mice used in the animal experiment were not assigned to groups using a randomization method. A single-blind analysis was adopted for the quantitative analysis in this experiment. We set the significance level (α) at 0.05 and power (1-β) at 80% to determine the sample size according to our preliminary experiments. No samples or animals were excluded from data analysis. The exact number of groups is included in figure legends.

### Ethics declarations

The use of animals and related experimental protocols in this study comply with the European Directive 2010/63/EU and have been approved by the Institutional Animal Care and Use Committee (IACUC) of Nanjing Drum Tower Hospital (Nanjing, China) (Approval No.: 2021AE01094). Except for analyzing the publicly available patient data in the GEO database, this manuscript does not involve any additional research related to human tissues or information.

### Serum lipid assay

At the experimental endpoint, mice were anesthetized with 5% isoflurane in Matrx Animal Aneathesia Ventilator System (VMR, MIDMARK Corporation, Torrance, CA, USA) before collecting the blood. Serum triglyceride (TG), total cholesterol (TC), low-density-lipoprotein cholesterol (LDL-C), and high-density-lipoprotein cholesterol (HDL-C) of mouse were measured by automatic clinical biochemical analysis system (Beckman coulter AU5421; USA).

### Oil red O staining

In order to assess atherosclerosis lesions from aorta arch to abdominal aortic bifurcation, the whole aorta of ten mice in each group were harvested after euthanasia. After carefully stripped the peripheral fat of the aorta, the whole aorta was stained with oil red O (Cat#01391; Sigma-Aldrich) for 30 min at 37 ^°^C and then washed with 60% isopropanol for 5 min. Images of whole aorta were captured by digital camera D7500 (Nikon; Tokyo, Japan).

The proximal aorta attached to heart was harvested to evaluate the size of atherosclerotic lesion in aortic root. And then it was embedded in optimum cutting temperature (OCT) compound after 24 h fixation in 4% paraformaldehyde. Next, serial 6-μm-thickness sections were transversely cut by Lecia CM1950 (Leica Biosystems, Wetzlar, Germany). The tissue sections were stained by oil red O for 25 min and then stained with hematoxylin (Cat#G1004; Wuhan, Servicebio) for 20 s, respectively. Then, the sections were immediately washed under running water for 2 min. The images of atherosclerotic plaques were quantified by Image J software after capturing by Olympus optical microscope.

### Hematoxylin-Eosin and MASSON staining

Hematoxylin-Eosin and Masson staining was performed using the Hematoxylin-Eosin (HE) (Cat#G1120) Stain Kit and Masson’s Trichrome Staining Kit (Cat#G1340) from Beijing Solarbio Science & Technology Co., Ltd. For analyzing the HE staining results of each mouse, 5 sections were finally selected (with an interval of 42 μm between every two sections and a total span of 210 μm), and their mean value was calculated as the final data for analysis. All experimental operations were strictly conducted in accordance with the kit’s instruction manual.

### Cultivation of primary bone marrow-derived macrophages (BMDM)

The primary BMDM was cultivated according to previous literature [28]. Briefly, the mice were sacrificed using isoflurane inhalation in a rodent euthanasia device and subsequent cervical dislocation. The skin of the mouse was disinfected with 70% alcohol solution. The skin and muscles were removed from the legs down to the hip bone. Then, the tibia and femur were isolated. And the epiphysis was cut and the bone marrow was rushed into centrifuge tube by PBS. Next, the suspension was filtrated with a 70 μm cell strainer in another centrifuge tube and centrifuged. Finally, the cell pellet was re-suspended to a 10 cm dish by using 10 ml RPMI 1640 medium (Cat#11875085; Gibco) containing 10% FBS, 2-Me, 2 mM sodium pyruvate (Cat#11360070; Gibco), 1 mM L-glutamine (Cat#25030081; Gibco), and non-essential amino acids (Cat#11140050; Gibco). After 3 days, the dish was gently shaken to spin down the cells that had not yet adhered, and then replaced the medium. After 5 days, medium was changed every other day. When the cells grew to 90% confluence, then were scraped with a spatula, and then re-inoculated in a 12-well plate for lipid accumulation assay. After 24 h, ox-LDL (Cat# 20605ES05; YESEN, Shanghai, China) was added to each well at 100 μg/ml and the incubation continued for 24 h for Nile red staining. The phenotypic identification results of mouse bone marrow-derived macrophages are shown in Supplementary Fig. 1 of the Supplementary materials.

### Cell cultures

Murine macrophage cell line Raw264.7 cells (TIB-71; ATCC, Shanghai, China) were maintained in complete DMEM medium (Cat#10566016; Gibco, Grand Island, NY, USA) supplemented with10% fetal bovine serum (FBS) (Gibco), 100 U/ml penicillin, and 100 μg/ml streptomycin (Cat#15140122; Sigma-Aldrich, St. Louis, MO, USA). Keep cells in a humidified incubator at 37 °C with 5% CO_2_. All cell lines were maintained in a mycoplasma-free state, and mycoplasma detection was performed on them using the fluorescence quantitative detection method and DAPI staining. The detailed results are shown in Supplementary Fig. 2.

HDAC6 shRNA and STAT3 shRNA were transduced into the Raw264.7 cell line using a lentiviral system. STAT3 siRNA was transfected into HDAC6 knockdown (KD) RAW264.7 cells using Lipofectamine™ RNAiMAX (Cat#13778100, Thermo Fisher). As negative controls, the PLKO.1-puro non-target shRNA control plasmid DNA from Sigma (Cat#SHC016, Sigma-Aldrich, St. Louis, MO, USA) and SignalSilence® Control siRNA (Cat#6568) were used; neither of these two reagents has known target genes, to assess interference efficiency by aligning target sequences. The sequences are provided in detail in Supplementary Table 3 of the Supplementary materials. All cell lines were maintained in a mycoplasma-free state. At the end of each cell experiment, cell samples were collected and tested for mycoplasma contamination using either the real-time fluorescence quantitative PCR method and DAPI staining. The results of mycoplasma detection for cultured cells are shown in Supplementary Fig. 2 of the Supplementary materials.

### Cholesterol uptake assay

Cells were harvested and seeded at a density of 2 × 10^4^ cells/mL in a 96-well-plate in a total volume of 100 µL medium per well. After 24 h incubation, the medium was removed, the cells were washed once with PBS, and then fresh medium containing 100 µM BODIPY-cholesterol and with or without STAT3 siRNA was added. Following incubation periods of 3, 6, and 12 h, the medium was transferred to another 96-well plate, and fluorescence intensity (Ex/Em = 505/515 nm) was measured using the Spark (Tecan, Switzerland) to quantify unabsorbed cholesterol. The monolayer cells were lysed overnight in 0.1 N NaOH. After centrifugation at 10,000 rpm for 5 min, the supernatant was collected, and the fluorescence intensity was detected as cholesterol uptake. The rate of cholesterol uptake was calculated as follows: cholesterol uptake rate = cholesterol uptake/(cholesterol uptake + cholesterol unabsorbed) × 100%.

### [1-^14^C]-palmitic acid beta-oxidation assay

The production of ^14^CO_2_ was used to assess the level of palmitic acid beta-oxidation as previously mentioned. Briefly, the cultured HDAC6 KD and control Raw264.7 cells were pre-incubated with glucose-free DMEM (Cat#A1443001; Invitrogen) for 30 min. Then, the 400 μM [^1-14^C]-palmitic acid was added and a piece of a wet no. 1 Whatman filter paper (American Chemical Society, USA) was alkalinized with 2 N sodium hydroxide (American MasterTech, New York, USA) and suspended within each flask. The palmitate oxidation of cells was terminated with 6 N hydrochloric acid (Cat#E484; Amresco, Washington, USA) in different time points (0, 6, 12, and 24 h). The production of ^14^CO_2_ was quantified by using scintillation counting.

### Nile red staining

Nile red, as the neutral lipid probe, was purchased from Sigma-Aldrich (Cat#N1142) and used to investigate the intracellular accumulation of lipids. The protocol of nile red staining as following: (1) 4% neutral paraformaldehyde was used to fix the treated cells for 15 min; (2) 0.5% Triton-X-100 (Beyotime Biotech, Jiangsu, Nanjing, China) was used to permeabilize the treated cells for 5 min; (3) nile red solution (1:10000 dilution from a saturated stock solution in acetone) was used to stain the treated cells for 30 min at room temperature; (4) 2-(4-Amidinophenyl)-6-indolecarbamidine dihydrochloride (DAPI) (Cat#ab104139; Abcam, Cambridge, MA, USA) was used to identify the nuclei; (5) immunostaining pictures were acquired by using the Leica DMi8 Confocal Microscope (Leica Microsystems, Wetzlar, Germany) at excitation wavelength 528 nm (green) and emission wavelength 636 nm (red).

### Immunoblotting

The protein samples were harvested with RIPA (Cat#P0013B; Beyotime Biotech), adding 1 mM protease, phosphatase inhibitor mix (Cat#P1045; Beyotime Biotech), and acetylase inhibitor (Cat#P1112; Beyotime Biotech). The concentration of protein of every sample was detected by BCA reagent (Cat#P0012; Beyotime Biotech). And the protein samples were boiled by adding 5 × SDS loading buffer for 5 min. Then, an equal quantity of protein of every sample were separated by SDS-PAGE. And then the protein was transferred to polyvinylidene difluoride (PVDF) membranes (Merck Millipore) from SDS-PAGE. Next, PVDF membranes were blocked with 5% skim milk for 2 h and then primary antibody was used to incubate the PVDF membranes at 4 °C for overnight. Finally, the secondary antibody was used to incubated for 1 h. The visible protein bands were detected by an enhanced chemiluminescence kit from Vazyme (Cat#E412-01). Full and uncropped immunoblot images are provided in the Supplementary material and the information of antibodies are provided in the Supplementary Table 4 of the Supplementary materials.

### Immunofluorescence staining

Firstly, the cryopreserved (−80 °C) tissue sections were fixed with 4% paraformaldehyde for 20 min after returning to room temperature. And then 0.5% Triton X-100 and 10% FBS were used to permeabilize and block those sections for 5 min and 20 min, respectively. Next, predetermined concentrations of primary antibodies were added dropwise to those sections overnight at 4 °C. The primary antibodies and the predetermined concentrations are provided in Supplementary Table 4 of the Supplementary materials. Finally, Alexa Fluor 488 conjugated secondary antibodies were used to incubate those sections and then DAPI was used to identify the nuclei. The images were captured the by using Leica DMi8 Confocal Microscope.

### Immunoprecipitation

The protein samples were harvested with NP40 (Cat#P0013F; Beyotime Biotech) adding 1 mM protease, phosphatase inhibitor mix, and acetylase inhibitor. The anti-Flag primary antibody was added to the cell lysate with gentle rocking overnight at 4 °C. Protein A or G agarose beads (Cat#sc-2003, Santa Cruz Biotechnology) were subsequently added for 5 h at 4 °C. Protein samples of immunoprecipitation was obtained by centrifugation at 3000 × *g* for 5 min, and then were washed by NP40 for 5 times. Finally, protein samples of immunoprecipitation were boiled by adding 2× SDS loading buffer of 25 μL for 5 min. The protein transfer, membrane blocking, antibody incubation, and protein detection steps were similar to those performed for immunoblotting.

### Statistical analysis

All experiments were performed with at least three replicates. All data were compiled statistics as means ± SEM (standard error of mean) by using GraphPad Prism 8 (GraphPad Software). The Shapiro–Wilk test was used to check the normality and group variance in all statistical data. For the comparison of two groups of data: the two-sided Student’s *t* test was used for data with a normal distribution, and the Mann–Whitney test was used for data without a normal distribution; for the differences in groupwise comparisons involving three or more groups (*n* > 2), they were determined by one-way analysis of variance (one-way ANOVA) combined with Tukey’s post-hoc test. The level of 5% was used to define the statistically significance threshold.

## Results

### HDAC6 negatively correlates with systemic lipid metabolism and monocyte expression in atherosclerosis

**Fig. 1:**
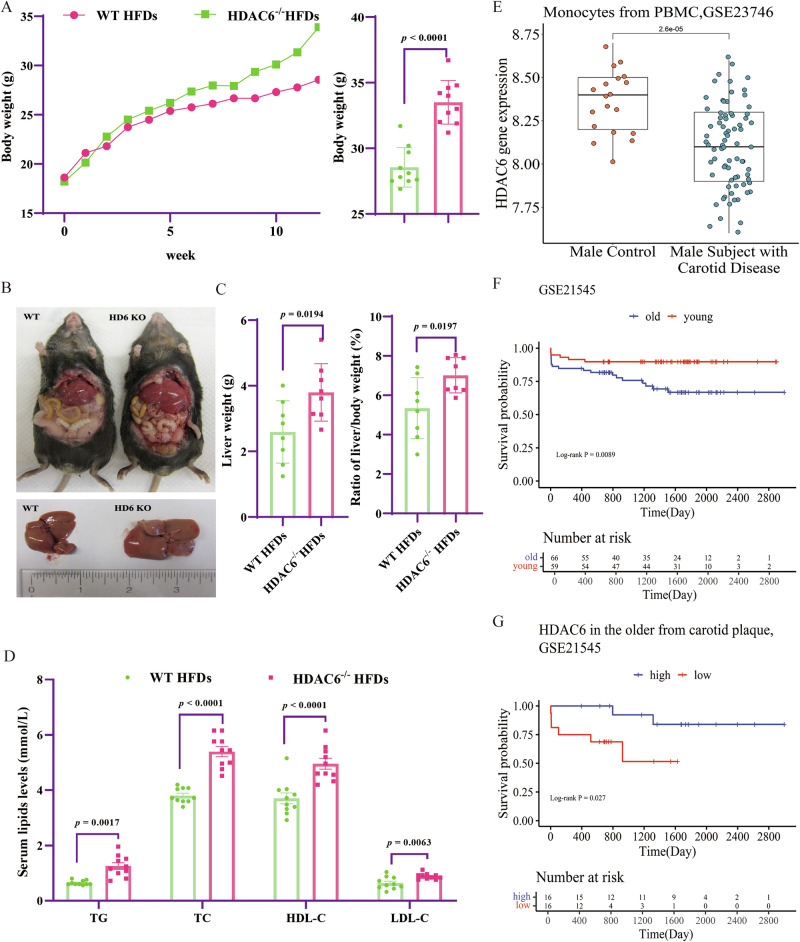
HDAC6 exhibits inverse association with systemic lipid metabolism and monocyte levels in atherosclerosis. **A** High fat food diets (HFDs) induced the body weight increased in *HDAC6*^−/−^ mice. Compared with WT group, the body weight in *HDAC6*^−/−^ mice was significantly higher at week 12 post HFDs feeding as showed in the right panel (*n* = 10, Student’s *t* tests). **B** The liver phenotype of WT and *HDAC6*^−/−^ mice at week 12 post-HFD feeding. **C** The liver weight (left) and ratio of liver/body weight (right) of WT and *HDAC6*^−/−^ mice at week 12 post HFD feeding (*n* = 8, Student’s *t* tests). **D** Serum TG, TC, HDL-C, and LDL-C levels of WT and *HDAC6*^−/−^ mice at week 12 post HFDs feeding. (*n* = 10, two-tailed Student’s *t* test). **E** The expression level of *HDAC6* in monocyte samples in patients with carotid atherosclerosis than normal control in male from the GSE23746 database. **F** Stratified analysis of the GSE21545 database based on age indicated that age may affect survival as a confounding factor. **G** Stratified by on the upper quartile of age, *HDAC6* showing the lower survival probability of low gene expression in patients older than 78 y from the GSE21545 database. The data are presented as the mean ± SEM. The values on the horizontal lines in the figure are the *P* values between the two groups. TG triglyceride, TC total cholesterol, HDL-C high-density-lipoprotein cholesterol, LDL-C low-density-lipoprotein cholesterol, PBMC peripheral blood mononuclear cell.

In systemic HDAC6 knockout (*HDAC6*^−/−^) mice fed a high-fat diet (HFD), body weight increased abnormally during the observation period compared to wild-type (WT) controls (Fig. 1A). Notably, the livers of *HDAC6*^−/−^ mice exhibited hypertrophy following HFD feeding relative to those of WT mice (Fig. 1B). Both the absolute liver weight and the liver-to-body weight ratio were significantly higher in *HDAC6*^−/−^ mice than in WT mice (Fig. 1C). Furthermore, after 12 weeks of HFD feeding, serum levels of total cholesterol (TC), triglycerides (TG), low-density-lipoprotein cholesterol (LDL-C), and high-density-lipoprotein cholesterol (HDL-C) were significantly elevated in *HDAC6*^−/−^ mice compared to the WT group (Fig. 1D). These findings indicate that HDAC6 deficiency disrupts lipid metabolism under conditions of excessive high-fat intake.

Given that dyslipidemia is a major risk factor for atherosclerosis, we investigated differences in HDAC6 expression between patients with atherosclerosis and matched controls. We analyzed two genomic expression profile datasets from the GEO database: GSE23746 and GSE21545. No identifiable participant information was available. Analysis of monocyte samples from the GSE23746 dataset revealed significantly lower HDAC6 expression in male patients with carotid atherosclerosis compared to normal controls (Student’s *t* test, *P* = 2.6 × 10^–5^) (Fig. 1E), suggesting an important role for monocyte HDAC6 expression in atherosclerosis. Further analysis of patients with atherosclerosis in the GSE21545 database, stratified by age using the upper quartile as a cutoff (Fig. 1F), demonstrated that in patients older than 78 years, lower HDAC6 expression in plaques significantly predicted adverse prognosis, as indicated by reduced survival probability (*P* = 0.0269) (Fig. 1G).

### *HDAC6* deficiency promotes lipid uptake and accumulation in macrophages

**Fig. 2:**
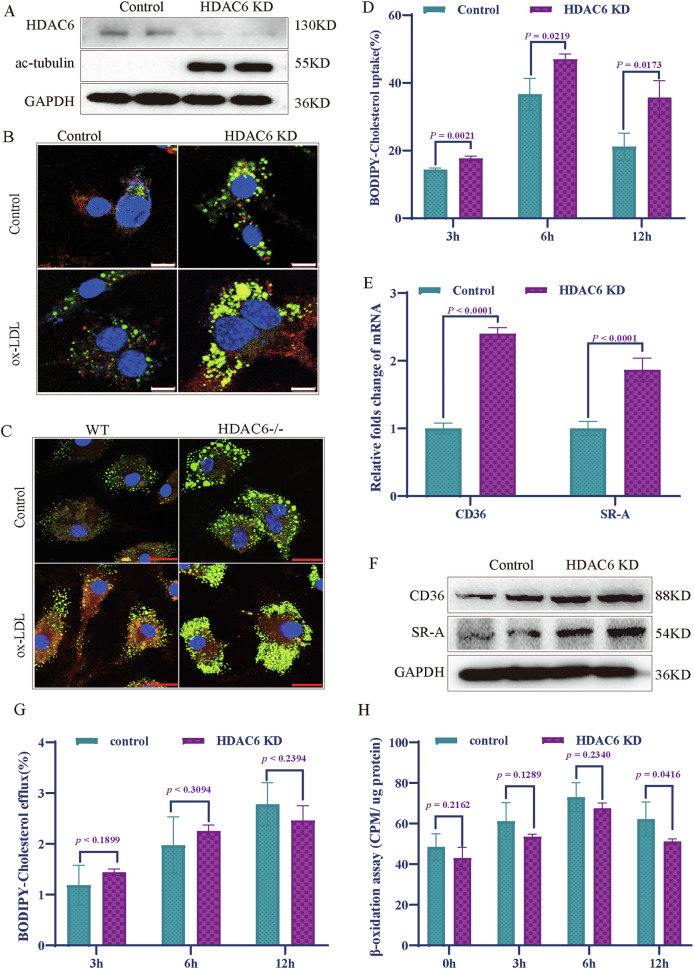
HDAC6 deficiency promotes lipid uptake and accumulation in macrophages. **A** The stable knockdown of HDAC6 in RAW264.7 cells was achieved by specific *HDAC6* shRNA. The HDAC6 and acetylation level of tubulin were determined by western blotting. **B** Nile red staining showed that HDAC6 deficiency significantly induced the intracellular lipid accumulation with or without treatment of ox-LDL (100 μg/ml), compared with control macrophages. **C** Nile red staining in primary BMDM cells from WT and *HDAC6*^*−/−*^ mice with 100 μg/ml ox-LDL treatment. Green: excitation at 528 nm; red: emission at 636 nm. **D** The level of cholesterol efflux was examined by BODIPY-cholesterol in control and *HDAC6* KD RAW264.7 cells (*n* = 3). **E**, **F** The transcriptional and protein expression levels of CD36 and SR-A in control and *HDAC6* KD RAW264.7 cells (*n* = 3). **G** The level of cholesterol efflux was examined by BODIPY-cholesterol in control and *HDAC6* KD RAW264.7 cells (*n* = 3). **H** The β-oxidation assay in control and *HDAC6* KD RAW264.7 cells by using palmitic acid labeled with carbon isotope [^1-14^C] (*n* = 3). Bar scale (white) = 8 μm, Bar scale (red) = 10 μm. HDAC6 histone deacetylase 6, ox-LDL oxidized low-density lipoprotein, BMDM bone marrow-derived macrophages.

To investigate the impact of *HDAC6* deficiency on macrophage lipid metabolism, we generated *HDAC6* knockdown (KD) RAW264.7 cells using short hairpin RNA (shRNA). Successful silencing of *HDAC6* was confirmed by elevated levels of acetylated tubulin (Fig. 2A). Nile red staining revealed that *HDAC6* KD induced significant lipid accumulation in RAW264.7 cells even under normal culture conditions without the addition of ox-LDL. Furthermore, treatment with 100 μg/ml ox-LDL led to markedly enhanced lipid accumulation in *HDAC6* KD cells compared to control cells (Fig. 2B). Similarly, primary bone marrow-derived macrophages (BMDMs) from *HDAC6*^−^^/^^−^ mice showed significantly greater lipid accumulation than wild-type (WT) BMDMs after treatment with 100 μg/ml ox-LDL (Fig. 2C). These findings indicate that *HDAC6* deficiency promotes excessive lipid uptake by macrophages, thereby facilitating their transformation into foam cells.

Given that lipid uptake, efflux, and β-oxidation are all involved in intracellular lipid droplet formation, we examined the effect of HDAC6 on these processes. Our results demonstrated that *HDAC6* deficiency significantly enhanced lipid uptake compared to controls (Fig. 2D). Consistently, both mRNA and protein levels of key lipid uptake receptors CD36 and SRA (scavenger receptor A) were significantly upregulated in *HDAC6*-deficient macrophages (Fig. 2E, F). In contrast, HDAC6-deficient macrophages exhibited no significant changes in lipid efflux; while β-oxidation was only significantly reduced at the 12-hour time point compared to controls (Fig. 2G, H).

### HDAC6 deacetylase dysfunction regulates STAT3 K685 deacetylation

**Fig. 3:**
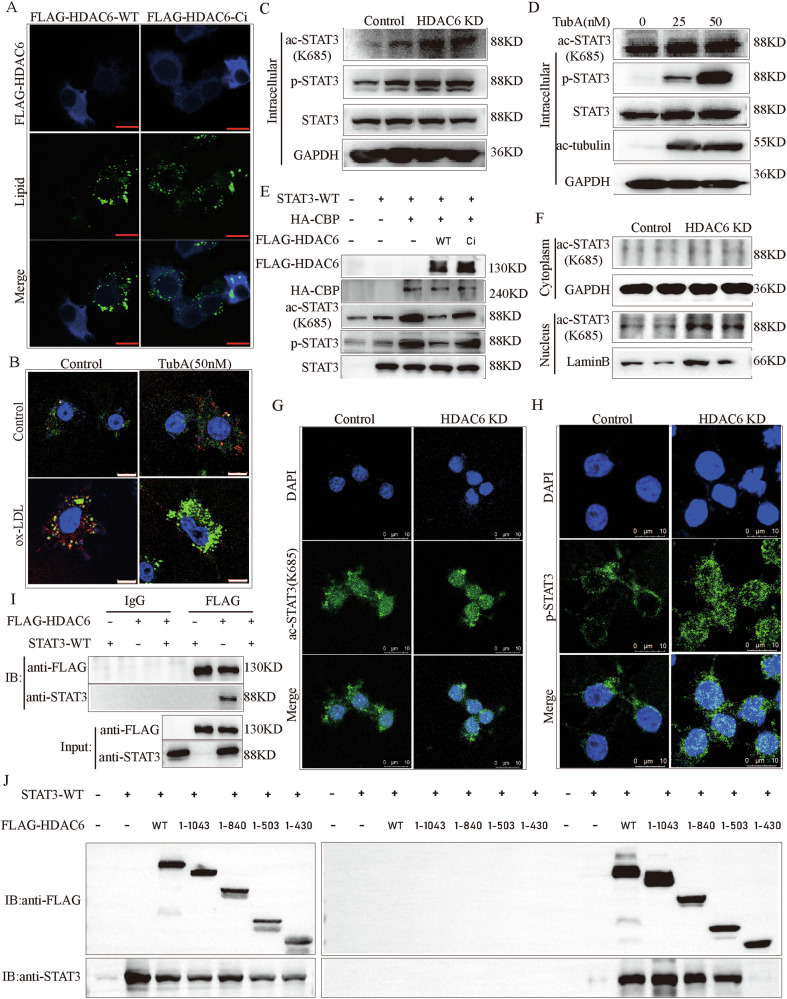
HDAC6 regulated STAT3 K685 deacetylation. **A** Overexpression of HDAC6 markedly blunted intracellular lipid accumulation in *HDAC6* KD RAW264.7 cells (blue: the expression of human HDAC6 gene, green: lipid droplets). **B** Nile red staining in Raw264.7 cells with TubA or with ox-LDL treatment. **C** The levels of ac-STAT3(K685) and p-STAT3 in intracellular in HDAC6 KD Raw264.7 cells compared with control Raw264.7 cells. **D** The levels of ac-STAT3(K685) and p-STAT3 in intracellular in Raw264.7 cells with Tubastatin A (TubA), a specific inhibitor that inhibits the deacetylase activity of HDAC6. **E** The effect of HDAC6 or HDAC6 with inactivated deacetylated functional on the activation of STAT3 by CBP. **F** The levels of ac-STAT3(K685) and p-STAT3 in intracellular, cytoplasmic, and nuclear in HDAC6 KD Raw264.7 cells compared with control Raw264.7 cells. **G**, **H** Immunofluorescence detected the ac-STAT3(K685) and p-STAT3 localization in HDAC6 KD compared to Control Raw264.7 cells. **I** The interaction between HDAC6 and STAT3 was identified by immunoprecipitation. **J** The interaction between different length of HDAC6 and full length STAT3 was identified by immunoprecipitation. STAT3 signal transducer and activator of transcription 3, p-STAT3 phosphorylated STAT3, ac-STAT3 acetylated STAT3, CBP cyclic AMP response element-binding protein, NES nuclear export signal, CD catalytic domain, DMB dynein-motor binding domain, SE14 serine glutamic acid tetrapeptide motif, ZnF-UBP Ubiquitin binding zinc finger domain.

HDAC6, a unique class IIb histone deacetylase, contains two deacetylase domains and a C-terminal ubiquitin-binding zinc finger domain (ZnF-UBP) that binds to ubiquitinated protein aggregates and targets misfolded proteins for lysosomal degradation [29]. To identify which functional domain of HDAC6 mediates the lipid accumulation induced by its deficiency in macrophages, we transiently transfected plasmids encoding wild-type HDAC6 (HDAC6-WT) or a deacetylation-inactive mutant (HDAC6-Ci) into HDAC6 knockdown (KD) RAW264.7 cells. Lipid droplet accumulation was then evaluated following treatment with 100 μg/ml ox-LDL (Fig. 3A; blue: HDAC6 expression, green: lipid droplets). Results showed that overexpression of HDAC6 inhibited lipid droplet accumulation in HDAC6 KD cells, whereas HDAC6-Ci failed to suppress lipid droplet formation. Similarly, Tubastatin A (TubA), a specific inhibitor of HDAC6 deacetylase activity, also promoted lipid accumulation upon ox-LDL treatment (Fig. 3B).

Phosphorylated STAT3 (p-STAT3) signaling has been shown to promote foam cell formation in macrophages[30]. Notably, beyond phosphorylation, acetylation at K685 (ac-STAT3(K685)) is also crucial for the formation of stable STAT3 dimers that effectively bind DNA and transcribe target genes [31]. However, whether HDAC6 inhibition regulates STAT3 K685 acetylation, and whether STAT3 acetylation directly contributes to foam cell formation remain unexplored. Therefore, we measured intracellular levels of ac-STAT3(K685) and p-STAT3 levels in HDAC6 KD versus control RAW264.7 cells. HDAC6 deficiency significantly increased both ac-STAT3(K685) and p-STAT3 levels (Fig. 3C). Treatment with TubA similarly elevated ac-STAT3(K685) and p-STAT3 levels in RAW264.7 cells (Fig. 3D).

To validate the regulatory role of HDAC6 in STAT3 acetylation and phosphorylation, we co-transfected plasmids encoding STAT3-WT, CBP, and either HDAC6-WT or HDAC6-Ci into 293T cells. As previously reported [32], CBP significantly upregulated ac-STAT3(K685) levels. HDAC6-WT, but not HDAC6-Ci, effectively blocked this CBP-induced STAT3 acetylation (Fig. 3E). Next, we analyzed ac-STAT3(K685) levels in cytoplasmic and nuclear fractions of HDAC6 KD versus control RAW264.7 cells (Fig. 3F). Nuclear ac-STAT3(K685) levels were significantly increased in HDAC6 KD cells, while cytoplasmic levels showed no significant difference. Immunofluorescence further confirmed stronger nuclear localization signals for both ac-STAT3(K685) and p-STAT3 in HDAC6 KD cells compared to controls (Fig. 3G, H).

Co-immunoprecipitation experiments in 293T cells co-transfected with STAT3-WT and Flag-HDAC6-WT plasmids revealed a direct interaction between HDAC6 and STAT3 (Fig. 3I). To map the HDAC6 domain required for interaction with STAT3, we co-expressed STAT3-WT with various FLAG-tagged HDAC6 truncation mutants (amino acids 1-1215 [full-length], 1-1043, 1-840, 1-503, or 1-430) in 293T cells. STAT3 co-immunoprecipitated with full-length HDAC6 (1-1215) and mutants 1-1043, 1-840, and 1-503, but not with the 1-430 fragment (Fig. 3J). This indicates that the catalytic domain 2 (CD2) of HDAC6 is necessary for binding STAT3.

### HDAC6 deacetylase inactivation promotes STAT3/CD36/SR-A-mediated lipid uptake

**Fig. 4:**
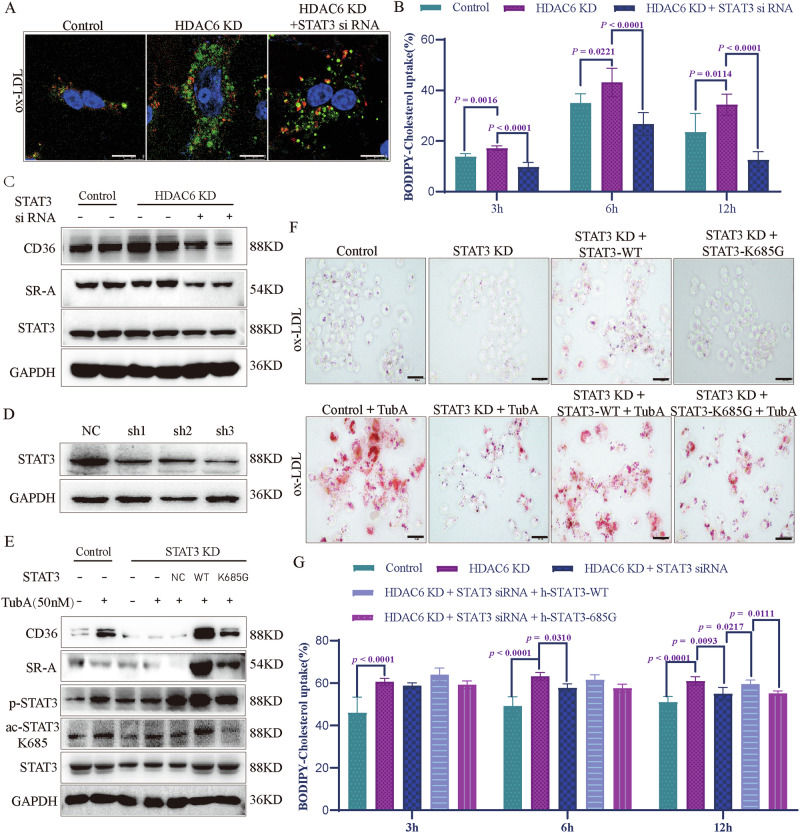
HDAC6 deficiency induced lipid accumulations by acetylation of STAT3 K685 in macrophages. **A** The effect of STAT3 siRNA on lipid accumulation in HDAC6 KD RAW264.7 cells with 100 μg/ml ox-LDL treatment. siRNA of STAT3 significantly inhibited the accumulation of lipid droplets in HDAC6 KD RAW264.7 cells. Bar scale (white) = 8 μm; **B** The level of cholesterol uptake was examined by BODIPY-cholesterol in control and *HDAC6* KD RAW264.7 cells with or without STAT3 siRNA (*n* = 3). **C** The expression of CD36 and SR-A in control and *HDAC6* KD RAW264.7 cells with or without STAT3 siRNA. **D** The interference efficiency of 3 different shRNA interference sequences were detected in RAW264.7 cells. **E** The levels of p-STAT3, ac-STAT3(K685), CD36, and SR-A after overexpression of wide type STAT3 protein or STAT3 protein of K685G in *STAT3* KD RAW263.7 cells after being treated by 50 nM TubA. **F** Oil red staining of lipid in overexpression of wide type STAT3 protein or STAT3 K685G in *STAT3* KD RAW263.7 cells after being treated by TubA (50 nM) and ox-LDL (100 μg/ml). Bar scale (black) = 100 μm. **G** The level of cholesterol uptake was examined by BODIPY-cholesterol after overexpressing the h-STAT3-WT or 685 G in *HDAC6* KD RAW264.7 cells with STAT3 siRNA. The data are presented as the mean ± SEM. The values on the horizontal lines in the figure are the *P* values between the two groups.

To determine whether STAT3 activation contributes to HDAC6 deficiency-induced lipid accumulation in macrophages, we silenced STAT3 expression using siRNA in HDAC6 KD RAW264.7 cells. STAT3 siRNA significantly suppressed lipid droplet accumulation in HDAC6 KD macrophages (Fig. 4A) and also inhibited cholesterol uptake, as well as the expression of CD36 and SR-A (Fig. 4B, C). We then established stable STAT3 KD RAW264.7 cells using three distinct shRNA sequences (Fig. 4D). These cells were transfected with plasmids expressing either wild-type STAT3 (STAT3-WT) or a lysine 685-to-glycine mutant (STAT3-K685G, an acetylation-deficient mutant) and treated with 50 nM TubA to inhibit HDAC6 activity. Compared to control cells, endogenous STAT3 deficiency significantly reduced the expression of CD36 and SR-A. Overexpression of STAT3-WT markedly upregulated CD36 and SR-A expression in 50 nM TubA-treated STAT3 KD cells. In contrast, overexpression of STAT3-K685G had minimal impact on CD36 and SR-A expression under the same conditions (Fig. 4E).

Next, we assessed lipid droplet accumulation using Oil Red O staining after treatment with 100 μg/ml ox-LDL treatment (Fig. 4F). Endogenous STAT3 deficiency significantly inhibited lipid droplet accumulation compared to controls. Overexpression of STAT3-WT largely reversed this inhibitory effect in STAT3 KD cells. Notably, compared to STAT3 KD cells expressing STAT3-WT (STAT3 KD/STAT3-WT), STAT3 KD cells expressing STAT3-K685G (STAT3 KD/STAT3-K685G) exhibited reduced lipid droplet accumulation following 50 nM TubA treatment. Finally, we investigated the role of STAT3 K685 acetylation in regulating macrophage cholesterol uptake upon HDAC6 inhibition (Fig. 4G). Overexpression of STAT3-WT increased cholesterol uptake in STAT3 siRNA-treated cells, whereas overexpression of STAT3-K685G did not.

### HDAC6 deficiency aggravates atherosclerotic lesions in *ApoE*^−/−^ mice

**Fig. 5:**
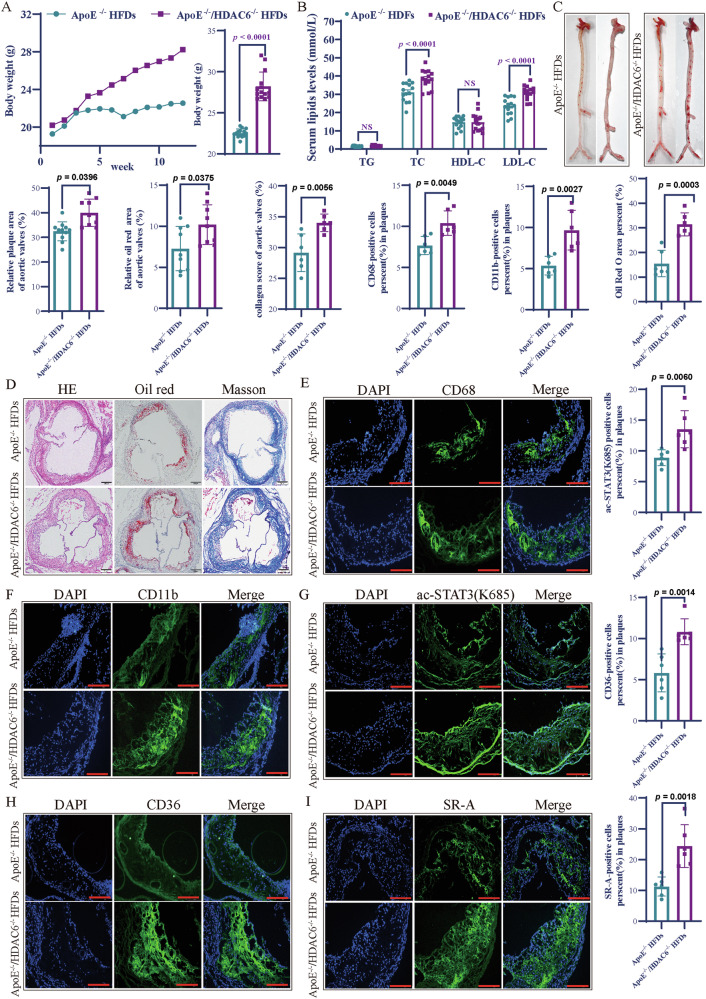
HDAC6 deficiency aggravates atherosclerotic lesions in ApoE-/- mice. **A** *ApoE*^−/−^/*HDAC6*^−/−^ mice had markedly higher body weight than *ApoE*^−/–^ after feeding with HFD. Compared with *ApoE*^−/−^ mice, the body weight in *ApoE*^−/−^/*HDAC6*^−/−^ mice was significantly higher at week 12 as shown in the right panel (*n* = 14 to each group, two-tailed Student’s *t* tests). **B** Serum HDL-C, TG, LDL-C, and TC in SKO and DKO mice at week 12 post HFD feeding. (*n* = 14 to each group, two-tailed Student's *t* test). **C** The oil red O staining of the atherosclerosis lesions from aorta arch to abdominal aortic bifurcation in *ApoE*^−/−^ and *ApoE*^−/−^/*HDAC6*^−/−^ mice (*n* = 6 to each group, two-tailed Student’s *t* tests). The result showed that HDAC6 deficiency promoted the more lipid accumulations in the arterial intima of *ApoE*^−/−^/*HDAC6*^−/−^ than *ApoE*^−/−^ mice. **D** The staining of H&E (*ApoE*^−/−^: *n* = 11; *ApoE*^−/−^/*HDAC6*^−/−^: *n* = 9), oil red O (*n* = 9 to each group), and MASSON (*n* = 6 to each group) of horizontal aortic root sections. The results showed that there were more atherosclerotic plaques, oil red O positive areas and collagen deposition in *ApoE*^−/−^/*HDAC6*^−/−^ mice than *ApoE*^−/−^ mice (two-tailed Student’s *t* tests). **E**, **F** Immunofluorescent staining showed there were more CD11b-positive and CD68-positive macrophages infiltration in aortic atherosclerotic plaques of *ApoE*^−/−^/*HDAC6*^−/−^ compared with *ApoE*^−/−^ mice (*n* = 6 to each group). Bar scale (blank) = 200 μm. Bar scale (wright) = 100 μm. H&E hematoxylin and eosin. **G**–**I** Immunofluorescent staining showed there were more ac-STAT3(K685)-positive, CD36-positive, and SR-A-positive (*n* = 6 to each group) macrophage infiltrations in aortic atherosclerotic plaques of *HDAC6*^−/−^/*ApoE*^−/−^ compared with *ApoE*^−/−^ mice. The data are presented as the mean ± SEM. The values on the horizontal lines in the figure are the *P* values between the two groups.

The occurrence and progression of atherosclerosis are closely linked to abnormal lipid metabolism and excessive lipid accumulation. To validate the role of HDAC6 in atherosclerosis, we crossbred *HDAC6*^−/−^ mice with *ApoE*^−/−^ mice to generate double-knockout (*ApoE*^−^^/−^/*HDAC6*^−^^/−^) mice. Mouse genotyping was confirmed via PCR and agarose gel electrophoresis (Supplementary Fig. 3A of the Supplementary materials). We monitored the changes of body weight and serum lipids in *ApoE*^−/−^/*HDAC6*^−^^/−^ mice after high- fat diet feeding during the observation period. Results showed that the *ApoE*^−/−^/*HDAC6*^−/−^ mice had higher body weights than *ApoE*^−/−^ mice (Fig. 5A). Additionally, compared with *ApoE*^−/−^ mice, the serum LDL-C and TC levels were significantly elevated in *ApoE*^−/−^/*HDAC6*^−/−^ mice, while there were no significant differences in serum TG and HDL-C levels between the two groups (Fig. 5B). However, in contrast to the WT group, high-fat diet did not induce lipid accumulation in the aortas of *HDAC6*^−/−^ mice (Supplementary Fig. 3B, C of the Supplementary materials).

Oil Red O staining of dissected whole aortas revealed substantially more severe atherosclerotic lesions in *ApoE*^−/−^/*HDAC6*^−/−^ mice compared to *ApoE*^−/−^ mice (Fig. 5C). Consistently, hematoxylin and eosin (H&E) staining showed larger lesion areas in the aortic roots of *ApoE*^−/−^/*HDAC6*^−/−^ mice. Oil red O staining of aortic root cross-sections further confirmed increased lipid accumulation in the double-knockout group. Additionally, Masson staining demonstrated enhanced collagen deposition within the aortic root lesions of *ApoE*^−/−^/*HDAC6*^−/−^ mice (Fig. 5D).

Immunostaining for CD68, a glycoprotein marker commonly used to identify macrophages[33], showed increased positivity in the aortic plaques of *ApoE*^−/−^/*HDAC6*^−/−^ mice compared to *ApoE*^−/−^ controls (Fig. 5E). Similarly, immunostaining for CD11b, a surface marker involved in monocyte/macrophage adhesion and migration, revealed significantly greater infiltration of CD11b-positive monocytes/macrophages into the atherosclerotic plaques of *ApoE*^−/−^/*HDAC6*^−/−^ mice (Fig. 5F). We also examined the expression levels of ac-STAT3(K685), CD36, and SR-A in aortic plaques from HFD-fed mice. Immunofluorescence staining demonstrated stronger signals for ac-STAT3(K685), CD36, and SR-A in the plaques of *ApoE*^−/−^/*HDAC6*^−^ mice relative to *ApoE*^−/−^ mice (Fig. 5G–I).

## Discussion

Members of the HDAC family exhibit diverse, and sometimes opposing, roles in atherosclerosis, regulating processes including inflammatory responses, vascular endothelial dysfunction, vascular smooth muscle cell proliferation, and plaque stability. Consequently, targeting specific HDAC isoforms with agonists or inhibitors represents a potential avenue for developing novel therapeutic strategies against atherosclerosis [34–36]. While evidence supporting existing HDAC agonists remains limited, studies using pan-HDAC inhibitors like trichostatin A (TSA), which targets Class I and II HDACs, have demonstrated increased plaque size in murine atherosclerosis models [25]. This highlights the critical importance of elucidating the distinct roles and regulatory mechanisms of individual HDAC members, as such knowledge is essential for rational development either pan-HDAC inhibitors or isoform-specific inhibitors to effectively treat atherosclerosis while minimizing adverse effects.

In this study, we analyzed data from the GEO database and identified significant differences in HDAC6 expression between patients with atherosclerosis and matched controls. Specifically, in the GSE23746 dataset, HDAC6 expression in monocytes was significantly lower in patients with carotid atherosclerosis compared to normal controls. Furthermore, data from the GSE21545 database indicated that lower HDAC6 expression in plaque correlated with reduced survival probability in patients over 78 years of age with carotid artery stenosis. Complementing these clinical observations, our in vivo findings demonstrated that HDAC6 deficiency markedly exacerbated atherosclerotic lesions in *ApoE*^−/−^ mice. This was evidenced by more severe lesions in dissected whole aortas, larger lesion areas and increased lipid accumulation in aortic roots, enhanced collagen deposition, and greater infiltration of monocytes/macrophages in *ApoE*^−/−^/*HDAC6*^−/−^ mice compared to *ApoE*^−/−^ controls. Although increased collagen deposition is often associated with plaque stabilization in atherosclerosis models, excessive accumulation may paradoxically elevate mortality risk under certain pathological conditions [37, 38].

Consistent with established knowledge, *ApoE*^−/−^ mice fed a high-fat diet (HFD) developed dyslipidemia (elevated TG, TC, and LDL) compared to wild-type (WT) mice [39]. Dysregulated lipid metabolism and adipokine imbalance are well-recognized links between obesity and atherosclerosis, with elevated LDL cholesterol promoting arterial lipid deposition and accelerating disease progression [40, 41]. Intriguingly, while our findings showed that HDAC6 deficiency in the *ApoE*^−/−^ background (*ApoE*^−/−^/*HDAC6*^−/−^ mice) led to increased body weight and higher serum TC and LDL-C levels, no significant lipid deposition was observed in the aorta or aortic valve of *HDAC6*^−/−^ mice alone. Both the absolute liver weight and the liver-to-body weight ratio were significantly higher in *HDAC6*^−/−^ mice than in WT mice. However, the deletion of HDAC6 did not significantly increase the liver weight or liver-to-body weight ratio in *ApoE*^−/−^ mice (Supplementary Fig. 4 of the Supplementary materials). This dissociation suggests that the dyslipidemia and lipid accumulation in monocyte-derived macrophages resulting from HDAC6 loss do not directly initiate atherosclerosis. TSA has been shown to exacerbate atherosclerosis without altering plasma lipid profiles [42]. SAHA, another pan-HDAC inhibitor targeting Class I and II HDACs, had no significant impact on body weight or serum HDL-C, TG, LDL-C, and TC in *ApoE*^−/−^ mice with HFD feeding [43]. Thus, while HDAC6 deficiency alone does not induce atherosclerosis under HFD conditions, its significant role in regulating lipid metabolism highlights its contribution to disease development in a susceptible background. This further underscores the complex and isoform-specific regulation of atherosclerosis by HDACs and the need for precise targeting to avoid unintended consequences.

To investigate the mechanisms underlying HDAC6’s role in macrophage lipid handling, we used primary bone marrow-derived macrophages (BMDMs) from WT and *HDAC6*^−/−^ mice treated with ox-LDL to simulate hyperlipidemic conditions. HDAC6 deficiency significantly promoted foam cell formation in WT macrophages in vitro, a finding corroborated in HDAC6-knockdown Raw264.7 cells, which exhibited striking lipid droplet accumulation upon ox-LDL treatment. As the primary cytoplasmic member of the HDAC family, HDAC6 has functions beyond deacetylation [14]; its C-terminal ZnF-UBP domain regulates the ubiquitin-proteasome system, contributing to intracellular protein homeostasis[29]. Using a deacetylation-deficient mutant, we confirmed that the induction of foam cell formation upon HDAC6 deletion is specifically attributable to the loss of its deacetylase activity. This role of HDAC6 in lipid droplet formation aligns with its documented functions in adipocytes and non-alcoholic fatty liver disease [26, 27].

Given the critical dependence of macrophage foam cell formation on HDAC6 deacetylase activity, identifying its key substrates is paramount for understanding its mechanisms and identifying potential therapeutic targets in atherosclerosis. STAT3, a transcription factor with a pivotal role in atherosclerosis, is a promising candidate. Its activation is tightly regulated during atherosclerosis, influencing macrophage pyroptosis, inflammation, lipid metabolism, and endothelial dysfunction [36]. In unstimulated cells, STAT3 is tightly controlled by negative regulators and resides inactive in the cytoplasm. Its activation, involving phosphorylation, acetylation, and dimerization, enables nuclear translocation. While the classical JAK/pSTAT3(Y705) pathway is well-known [44], acetylation enhances STAT3’s DNA binding, transactivation potential, and nuclear localization [45]. Although HDAC1, HDAC3, and HDAC10 have been demonstrated to be involved in inhibiting the acetylation of STAT3 [46–48], the role of HDAC6-mediated deacetylation of STAT3 (specifically at K685) in atherosclerosis remained unexplored. Our study revealed that HDAC6 deficiency upregulated K685 acetylation of STAT3 in macrophages in vitro. Conversely, HDAC6 overexpression inhibited CBP-induced STAT3 K685 acetylation. Immunofluorescence and immunoblotting analyses further demonstrated that HDAC6 deficiency enhanced the nuclear translocation and signaling of K685-acetylated STAT3. Critically, in vitro experiments validated that the induction of macrophage lipid deposition following HDAC6 deletion or pharmacological inhibition (using the specific inhibitor TubA) was mediated through STAT3 K685 acetylation, as evidenced by rescue experiments using STAT3 siRNA and K685 mutant plasmids. These findings elucidate the regulatory role and mechanism of intracellular HDAC6 in modulating STAT3 acetylation. Synergistic effects between STAT3 acetylation and phosphorylation have been reported. For example, A-485, a potent and specific p300/CBP inhibitor, disrupts both STAT3 acetylation at K685 and phosphorylation [49]. In vascular smooth muscle cells, inhibited the interaction between SIRT1 and STAT3 proteins promotes STAT3-K685 acetylation and STAT3-Y705 phosphorylation [50]. Consistent with these reports, our results showed that phosphorylated STAT3 levels in macrophages were significantly increased after inhibiting HDAC6 deacetylase function, and mutation of STAT3 at K685 affected STAT3 phosphorylation levels following HDAC6 deletion—further validating the cooperative regulatory mechanism of STAT3 phosphorylation and acetylation. However, some studies have reported a competitive relationship between these modifications: for example, inhibiting of HDAC1/2 simultaneously increases STAT3 acetylation and reduce its phosphorylation in human aortic endothelial cell [22], and inhibiting HDAC6 activity with the selective HDAC6 inhibitor ACY-1215 upregulates STAT3 acetylation while suppressing its phosphorylation in neurons [51]. Those findings indicate complex synergistic and competitive interactions between STAT3 phosphorylation and acetylation in different cellular or disease contexts.

Furthermore, we demonstrated that the HDAC6/acSTAT3(K685) axis directly participates in the transcriptional upregulation of CD36 and SR-A, thereby promoting ox-LDL uptake in macrophages. This mechanism is supported in vivo by the stronger immunofluorescence signals for ac-STAT3(K685), CD36, and SR-A in aortic atherosclerotic plaques of *ApoE*^−/−^/*HDAC6*^−/−^ mice compared to *ApoE*^−/−^ mice. Collectively, these results illuminate the role of the HDAC6/ac-STAT3(K685)/CD36/SR-A signaling axis in lipid-induced macrophage foam cell formation, primarily via enhanced lipid uptake. This pathway underlies the exacerbated lipid deposition, plaque enlargement, and increased monocyte/macrophage infiltration into the aortic wall in HDAC6 deficiency.

In this study, we demonstrated that HDAC6 KO, both alone and in combination with *ApoE*^−/−^, leads to significant metabolic disturbances and promotes macrophage-derived foam cell formation in mice. Furthermore, HDAC6 deficiency within an *ApoE*^−/−^ background exacerbated atherosclerotic lesions. However, the current model does not allow us to fully dissociate the specific effects of HDAC6 ablation from the overall impact of systemic metabolic derangement. To conclusively determine the monocyte/macrophage-specific contribution of HDAC6 deletion to atherosclerotic progression, future studies using an inducible, monocyte-specific HDAC6 KO model would be highly valuable. This limitation is acknowledged in the present work.

In summary, GEO database analysis identified significantly reduced HDAC6 expression in monocytes from carotid atherosclerosis patients, correlating with adverse prognosis in elderly patients. In vivo studies in *ApoE*^−/−^ mice demonstrated that *HDAC6* knockout exacerbates atherosclerotic lesions and macrophage infiltration within aortic valve lesions. We further established that the deacetylase function of HDAC6 is a key regulator of macrophage lipid metabolism, with the underlying mechanism involving HDAC6 modulating lipid uptake via the ac-STAT3(K685)/CD36/SR-A pathway in macrophages. Importantly, our results indicate that the effect of HDAC6 knockout on atherosclerosis contrasts with the reported protective role of HDAC9 knockout. This distinction provides crucial insights for the potential application of HDAC inhibitors in atherosclerosis treatment, emphasizing the need for isoform selectivity to avoid detrimental side effects.

## Supplementary information

Supplementary material

Supplemental Figure 1

Supplemental Figure 2

Supplemental Figure 3

Supplemental Figure 4

Supplemental Table 1

Supplemental Table 2

Supplemental Table 3

Supplemental Table 4

WB-uncropped

## Data Availability

The datasets GSE23746 and GSE21545 of atherosclerosis samples used in this study were all downloaded from the GEO database (https://www.ncbi.nlm.nih.gov/geo/). All other data are available from the corresponding author upon reasonable request.
