# Supplementary material for: HDAC6 deficiency exacerbates atherosclerosis via STAT3-K685 acetylation-mediated CD36/SR-A upregulation in macrophages
Source: Cell Death Dis. 2025 Dec 24;17(1):135. doi: 10.1038/s41419-025-08344-y (PMC12848014; doi:10.1038/s41419-025-08344-y)
Supplement: Supplementary file 1 — Supplementary material [file 41419_2025_8344_MOESM1_ESM.docx]

**Supplemental Material**

**Contents**

**Ⅰ Supplemental Figures**

**Figure S1:** **Phenotypic Identification of Primary Bone Marrow-Derived Macrophages**

**Figure S2:** **Mycoplasma Detection in Cultured Cells**

**Figure S3: Systemic knockout of HDAC6 alone fails to induce the atherosclerotic phenotype in mice after 12 weeks of high-fat diet feeding**

**Figure S4: The changes of liver in *ApoE*^-/-^ and *ApoE*^-/-^/*HDAC6*^-/-^ mice after 12 weeks of high-fat diet feeding**

**Ⅱ Supplemental Tables**

**Table S1: Primer’s sequence for genetyping.**

**Table S2: PCR Protocol for Genotype Identification.**

**Table S3: Sequence of HDAC6/STAT3 shRNA or STAT3 siRNA.**

**Table S4: Details of the antibodies use.**

**Ⅰ Supplemental Figures**

**Figure S1**

**Figure S1. Phenotypic Identification of Primary Bone Marrow-Derived Macrophages (BMDMs) of Mice** (A) Flow Cytometry (FACS) Analysis of Cell Suspensions of Mouse Primary Bone Marrow-Derived Macrophages (BMDMs). Over 90% of the cells were positive for CD11b and F4/80, which are markers for murine macrophages (n = 3). (B) Fluorescent Labeling of Mouse Primary Macrophages with CD68. (n =3) (Bar scale = 50 μm).

**Figure S2**

**Figure S2. Detection of Mycoplasma in cell cultures** (A) Detection of Mycoplasmal DNA by Real-Time Fluorescence Quantitative Method. Yellow S-shaped amplification curve (number 1): Mycoplasma positive control，Ct value = 9; other amplification curves: Ct value > 35, which is much higher than the 9 cycles of the positive control, considered mycoplasma-negative. Different colors corresponding to numbers 2, 3, 4, 5, and 6 represent the shRNA control group, HDAC6 KD group, STAT3 KD group, WT-BMDMS group, and HDAC6 KO-BMDMs group, respectively (n = 3). (B) DAPI staining of DNA. DAPI labeled the DNA in the cell nuclei as expected; no fluorescent signals were observed in the cytoplasm (n = 3) (Bar scale = 10 μm).

**Figure S3**

**Figure S3. Systemic knockout of HDAC6 alone fails to induce the atherosclerotic phenotype in mice after 12 weeks of high-fat diet feeding** (A) The mouse genotyping was confirmed by PCR and then agarose gel electrophoresis. Lane 1 and 7 represented DNA marker, Lane 2 and 3 represented *ApoE* WT, Lane 4 and 5 represented *ApoE*^-/-^, Lane 6 represented heterozygote of *ApoE*; Lane 8 and 10 represented *HDAC6* WT, Lane 9 and 11 represented *HDAC6*^-/-^. (B) The oil red O staining of the atherosclerosis lesions from aorta arch to abdominal aortic bifurcation in WT and *HDAC6*^-/-^ mice (n = 4). (C) The staining of H&E horizontal aortic root sections (n = 4) (Bar scale = 100 μm).

**Figure S4**

**Figure S4. The changes of liver in *ApoE*^-/-^ and *ApoE*^-/-^/*HDAC6*^-/-^ mice after 12 weeks of high-fat diet feeding** (A and B) The liver weight and ratio of liver/body weight of *ApoE*^-/-^ and *ApoE*^-/-^/*HDAC6*^-/-^ mice at week 12 post HFD feeding (n = 14, Student’s t-tests).

**Ⅱ Supplemental Tables**

Table S1. Primer’s sequence for genetyping

Table S2. PCR Protocol for Genotype Identification

Table S3. Sequence of HDAC6/STAT3 shRNA, STAT3 siRNA, and primer sequence of CD36 and SRA

Table S4. Details of the antibodies used.
