# Supplementary figures and images for: HDAC6 deficiency exacerbates atherosclerosis via STAT3-K685 acetylation-mediated CD36/SR-A upregulation in macrophages
Source: Cell Death Dis. 2025 Dec 24;17(1):135. doi: 10.1038/s41419-025-08344-y (PMC12848014; doi:10.1038/s41419-025-08344-y)

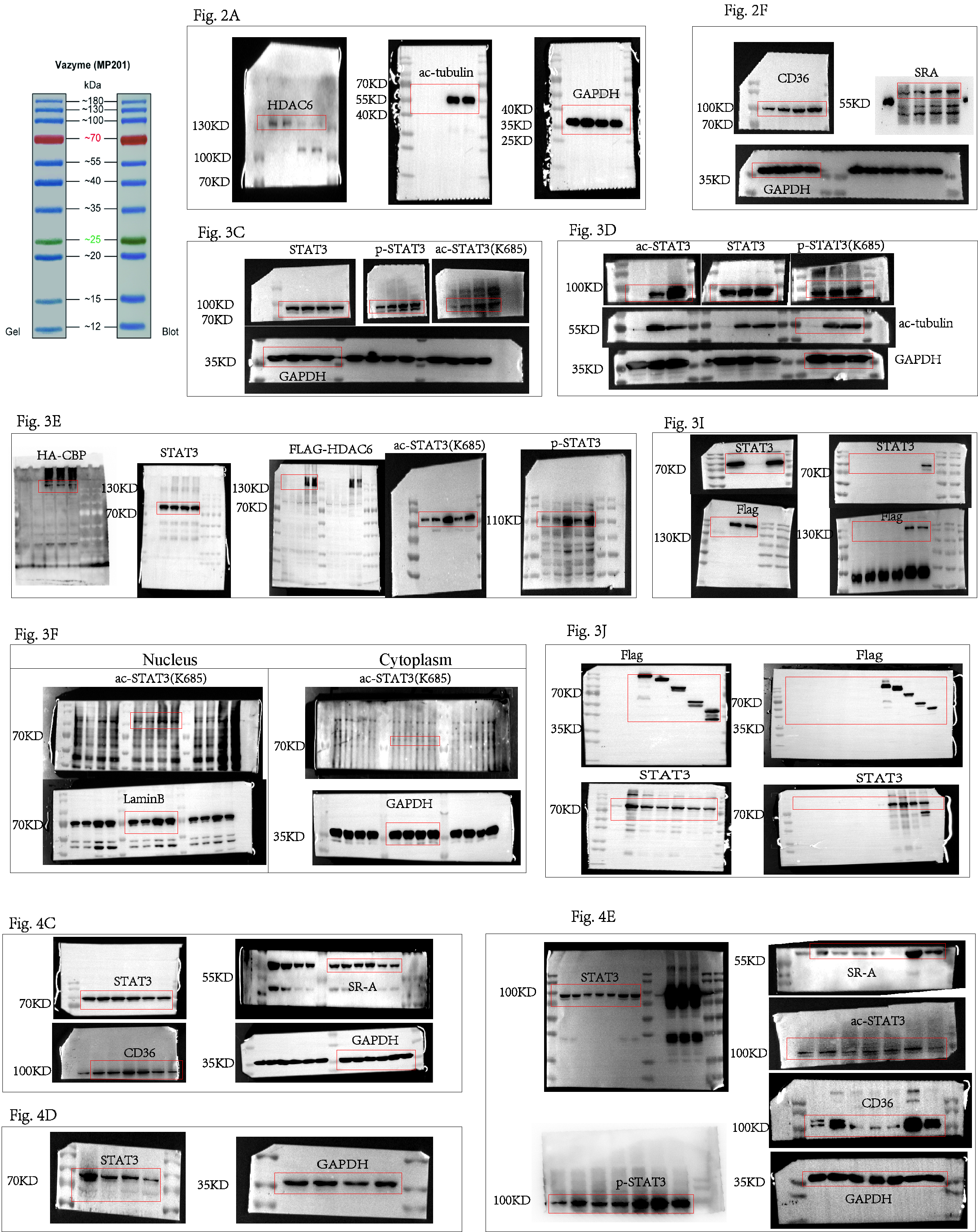

Supplement: Supplementary file 2 — Supplemental Figure 1 [file 41419_2025_8344_MOESM2_ESM.tif]

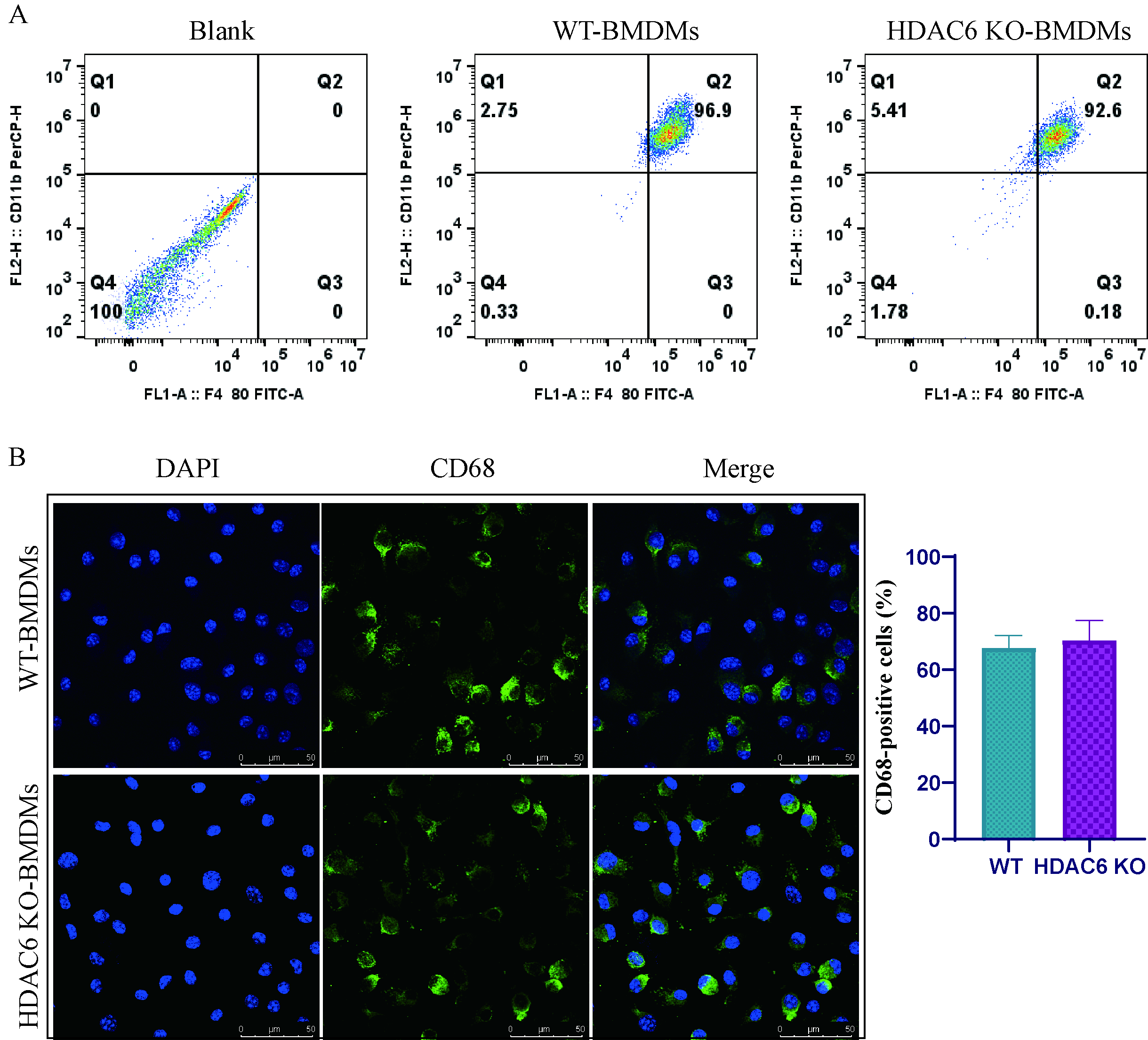

Supplement: Supplementary file 3 — Supplemental Figure 2 [file 41419_2025_8344_MOESM3_ESM.tif]

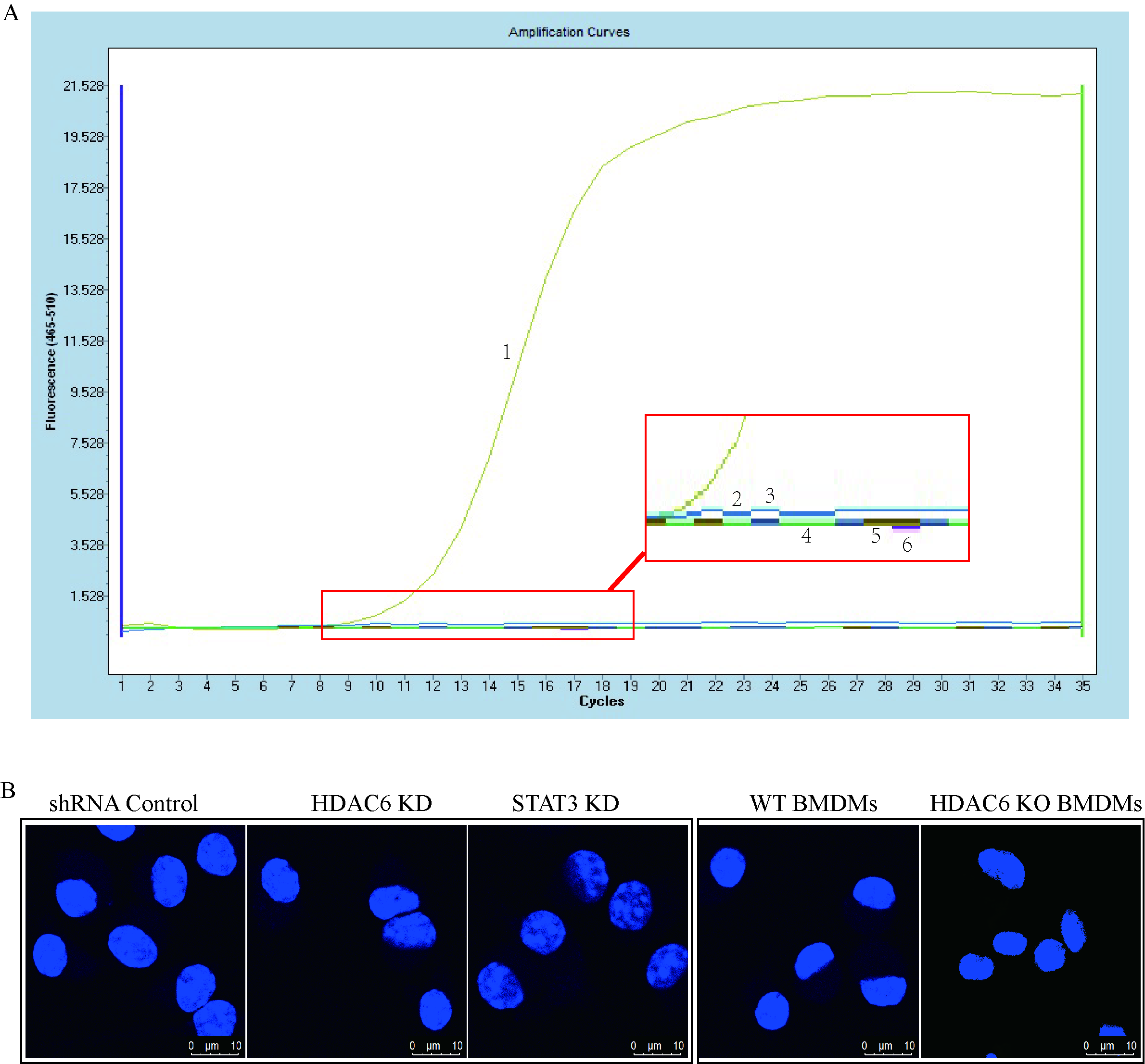

Supplement: Supplementary file 4 — Supplemental Figure 3 [file 41419_2025_8344_MOESM4_ESM.tif]

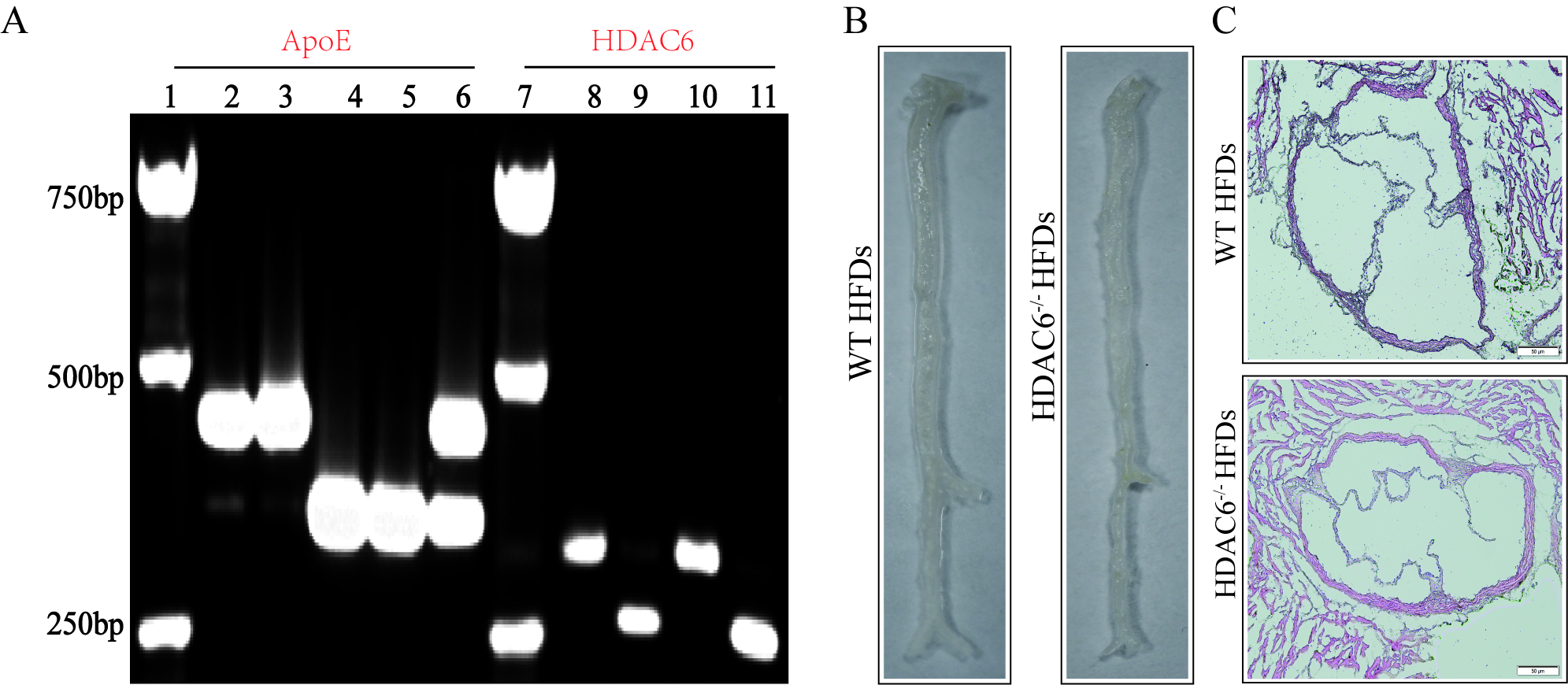

Supplement: Supplementary file 5 — Supplemental Figure 4 [file 41419_2025_8344_MOESM5_ESM.tif]

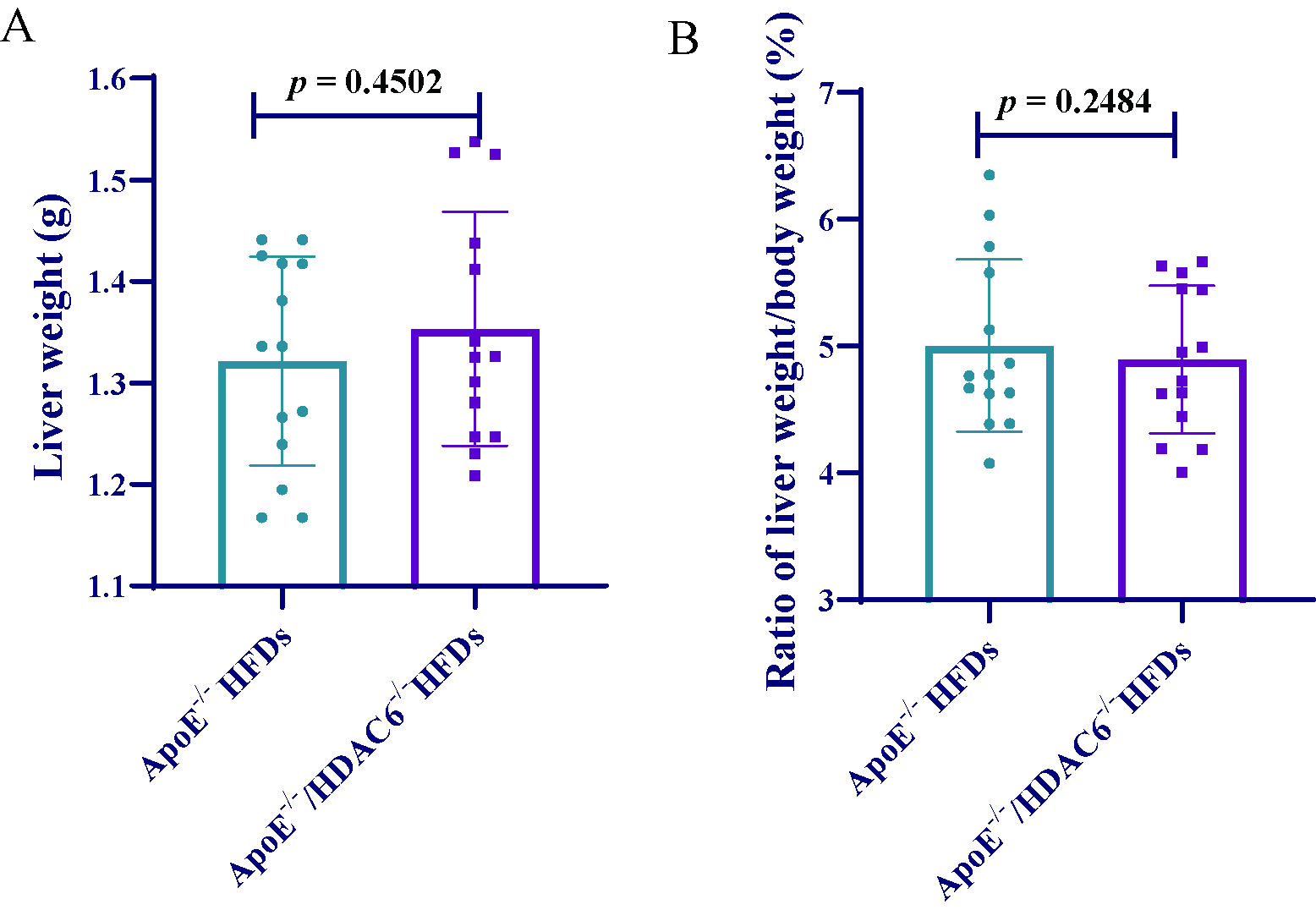

Supplement: Supplementary file 6 — Supplemental Table 1 [file 41419_2025_8344_MOESM6_ESM.tif]
