# Supplementary material for: HDAC6 deficiency exacerbates atherosclerosis via STAT3-K685 acetylation-mediated CD36/SR-A upregulation in macrophages
Source: Cell Death Dis. 2025 Dec 24;17(1):135. doi: 10.1038/s41419-025-08344-y (PMC12848014; doi:10.1038/s41419-025-08344-y)
Supplement: Supplementary file 7 — Supplemental Table 2 [file 41419_2025_8344_MOESM7_ESM.docx]

| Gene name | Sequence |
| --- | --- |
| ApoE | Forward primer: TGC CTA GTC TCG GCT CTG AAC TAC |
|  | Reverse primer: CAA CCT GGG CTA CAC ACT AAT TGA G |
| HDAC6 | zeo-1(CCA TGA CCG AGA TCG GCG AGC A) |
|  | zeo-3(CGT GAA TTC CGA TCA TAT TCA AT) |
|  | Lnt-9(CTG GTT CGT CTG AAG ACA) |
|  | Exo-10(GTG GAC CAG TTA GAA GCC) |
