# Supplementary material for: HDAC6 deficiency exacerbates atherosclerosis via STAT3-K685 acetylation-mediated CD36/SR-A upregulation in macrophages
Source: Cell Death Dis. 2025 Dec 24;17(1):135. doi: 10.1038/s41419-025-08344-y (PMC12848014; doi:10.1038/s41419-025-08344-y)
Supplement: Supplementary file 8 — Supplemental Table 3 [file 41419_2025_8344_MOESM8_ESM.docx]

| Gene | Temperature | Time | Cycle |
| --- | --- | --- | --- |
| ApoE | 95°C | 5 min | 1X |
|  | 95°C | 30 s | 35 |
|  | 58°C | 30 s |  |
|  | 72°C | 45 s |  |
|  | 72°C | 5 min | 1X |
|  | 4°C | hold |  |
| HDAC6 | 95°C | 3 min | 1X |
|  | 95°C | 30 s | 30 |
|  | 55°C | 30 s |  |
|  | 72°C | 1min 30s |  |
|  | 72°C | 10 min | 1X |
|  | 4°C | hold |  |
