# Supplementary material for: HDAC6 deficiency exacerbates atherosclerosis via STAT3-K685 acetylation-mediated CD36/SR-A upregulation in macrophages
Source: Cell Death Dis. 2025 Dec 24;17(1):135. doi: 10.1038/s41419-025-08344-y (PMC12848014; doi:10.1038/s41419-025-08344-y)
Supplement: Supplementary file 9 — Supplemental Table 4 [file 41419_2025_8344_MOESM9_ESM.docx]

| Primer name | Sequences |
| --- | --- |
| HDAC6 shRNA | 5′-GCT GAC TAC ATT GCT GCT TT-3′ |
| STAT3 shRNA-1 | 5'-TAG TGA AGA AGT CTA GCG TCT-3' |
| STAT3 shRNA-2 | 5'-CCA GTA CAT CAG CGA TAA ATT-3' |
| STAT3 shRNA-3 | 5'-TTC ACA GGG TCG ATG ATA TTG-3' |
| STAT3 siRNA | 5′-CAG GGU GAC AGA CAU GGC CUA-3′ |
| CD36 forward primer | 5′-GAT GAC GTG GCA AAA GAA CAG-3′ |
| CD36 reverse primer | 5′-TCC TCG GGG TCC TGA GTT AT-3′ |
| SRA forward primer | 5'-GCC AAC CTC ATG GAC ACA GA-3' |
| SRA reverse primer | 5'-AGA ATT TCC TGG CTT CCG G-3' |
