# Supplementary material for: HDAC6 deficiency exacerbates atherosclerosis via STAT3-K685 acetylation-mediated CD36/SR-A upregulation in macrophages
Source: Cell Death Dis. 2025 Dec 24;17(1):135. doi: 10.1038/s41419-025-08344-y (PMC12848014; doi:10.1038/s41419-025-08344-y)
Supplement: Supplementary file 10 — WB-uncropped [file 41419_2025_8344_MOESM10_ESM.docx]

| Antibody | Supplier | Catalog number | Dilution |
| --- | --- | --- | --- |
| Goat Anti-Rabbit IgG (H+L) HRP | Bioworld Biotech | BS13278 | IB: 1:40000 |
| Goat Anti-Mouse IgG (H+L) HRP | Bioworld Biotech | BS12478 | IB: 1:40000 |
| GAPDH | Servicebio^TM^ | GB11002 | IB: 1:4000 |
| HDAC6 | invitrogen | PA1-25455 | IB: 1:1000 |
| Ac-tubulin | Proteintech^TM^ | 66200 | IB: 1:1000 |
| CD36 | Proteintech^TM^ | 18836-1-AP | IB: 1:2000 |
| CD36 | Abcam | Ab252922 | IF: 1:500 |
| SRA | Proteintech^TM^ | 17858-1-AP | IB: 1:1000  IF: 1:500 |
| STAT3 | CST | 9139 | IB 1:1000 |
| p-STAT3 | Beyotime Biotech | AF1276 | IB: 1:1000 |
| Ac-STAT3 | CST | 2523 | IB: 1:1000  IF: 1:500 |
| Lamin B | Proteintech^TM^ | 12987-1-AP | IB: 1:1000 |
| Flag | Proteintech^TM^ | 80010-1-RR | IB: 1:1000 |
| HA | Proteintech^TM^ | 81290-1-RR | IB: 1:1000 |
| IgG | Proteintech^TM^ | 30000-0-AP | IB: 1:1000 |
| CD68 | Abcam | Ab53444 | IF: 1:500 |
| CD11b | Proteintech^TM^ | 66519-1 | IF: 1:500 |
| Alexa Fluor 488 conjugated secondary(anti-rabbit) | invitrogen | A11034 | IF: 1:500 |
| Alexa Fluor 488 conjugated secondary(anti-mouse) | invitrogen | A11011 | IF: 1:500 |
| Alexa Fluor 488 conjugated secondary (anti-Rat) | invitrogen | A-11006 | IF: 1:1000 |
